# Supplementary material for: Diagnostic Performances of Urinary Methylmalonic Acid/Creatinine Ratio in Vitamin B12 Deficiency
Source: J Clin Med. 2020 Jul 22;9(8):2335. doi: 10.3390/jcm9082335 (PMC7466029; doi:10.3390/jcm9082335)
Supplement: Supplementary file 1 [file jcm-09-02335-s001.pdf]

Supplementary Materials

Materials and Methods—Biological assays

Plasma Vitamin B12

The plasma vitamin B12 was collected in EDTA tubes and determined by immunoassay by competition, with direct chemiluminescence. The tests were carried out on an immunoanalytical system ADVIA Centaur® (Siemens Healthcare Diagnostics Inc. Tarrytown, NY 10591-5097 USA) with ADVIA Centaur VB12® reagents. The reference values considered as normal by the supplier ranged from 198 to 986 ng/L with a coefficient of variation of 1.3–4.1%.

Plasma Homocysteine

Blood was collected in EDTA tubes and samples were quickly (<1h) delivered to the laboratory immersed in ice and centrifuged, and supernatant was stored at −20 °C until extraction and analysis. Plasma was reduced with dithiothreitol after the addition of DL-homocysteine-D8 as an internal standard to monitor the extraction step. Proteins were then precipitated by acetonitrile + formic acid 0.1% + trifluoroacetic acid 0.05% and centrifuging. Samples were then analyzed by HPLC-MS/MS (Agilent 1200 Infinity Series, Agilent Technologies, Santa Clara, CA, USA, Triple Quad™ 4500, SCIEX, Framingham, MA, USA). Quantification was calibrated with homocysteine standard solutions ranging from 0 to 98.7 μM. Samples with concentration above the limit of the calibration curve (98.7 μM) were diluted. Two Clincheck® quality controls (Levels 1 & 2, LGC Ltd, Teddington, TW11 0LY, UK) were measured at the beginning and the end of each run. The coefficient of variation was of 1.5–1.6%.

Plasma Methylmalonic Acid

Blood samples were collected in lithium heparin tubes, quickly (<1h) centrifuged and plasma was stored at −20 °C until extraction and analysis. MMA was extracted from plasma by a methyl tert-butyl ether/phosphoric acid solution, after the addition of d3-MMA as an internal standard. After evaporation, dried extracts were butylated with 1-butanol/HCl and then evaporated again. Samples were then solubilized with acetonitrile/water 70/30 and analyzed by HPLC-MS/MS (Agilent 1200 Infinity Series, Agilent Technologies, Santa Clara, CA, USA, Triple Quad™ 5500, SCIEX, Framingham, MA, USA). Quantification was calibrated with MMA standard solutions ranging from 0 to 5 μM. Samples with concentration above the limit of the calibration curve (5 μM) were diluted. Two Clincheck® quality controls (Levels 1 & 2, LGC Ltd, Teddington, TW11 0LY, UK) were measured at the beginning and the end of each run. The coefficient of variation was of 2.6-5.0%.

Urinary Methylmalonic Acid

Urine samples were collected in sterile tubes and stored at −20 °C until extraction and analysis. The analysis method carried out allows the quantification of uMMA among a panel of urinary organic acids. Samples were diluted with NaCl 0.9% to the concentration of 1 mM creatinine, made up to 1 mL, and acidified with 200 μL HCl 2.4 N. 3 μL of a solution of 8 mM phenylbutyric acid were added as an internal standard to monitor the extraction step. Urinary organic acids were extracted three times with 2 mL of ethyl acetate. Organic phases were pooled and evaporated. Dried extracts were trimethyl-silylated by adding 100 μL of a BSTFA/pyridine solution and heating at 80 °C during 45 min. Samples were analyzed by GC-MS (QP 2010S, Shimadzu, Kyoto, Japan). Quantification was calibrated with dilutions of a standard solution of organic acids ranging from 0 to 800 μM. Two quality control levels (QC) (IQCS organic acids levels 1 and 2, ERNDIM, Manchester M13 9WL, United Kingdom) were measured at the beginning and the end of each run. Calibration solutions and QCs were processed with the same protocol as samples (coefficient of variation: 7.2–8.8%).
